# Supplementary material for: Integration of shared-pathogen networks and machine learning reveals the key aspects of zoonoses and predicts mammalian reservoirs
Source: Proc Biol Sci. 2020 Feb 5;287(1920):20192882. doi: 10.1098/rspb.2019.2882 (PMC7031665; doi:10.1098/rspb.2019.2882)
Supplement: Electronic supplementary notes and materials [file rspb20192882supp1.pdf]

Integration of shared-pathogen networks and machine learning reveal key aspects of zoonoses and predict mammalian reservoirs – Electronic Supplementary Materials

Maya Wardeh, Kieran J. Sharkey, Matthew Baylis

Corresponding author:

Maya Wardeh

Department of Epidemiology and Population Health, Institute of Infection and Global Health, University of Liverpool, Liverpool Science Park IC2 Building,

146 Brownlow Hill, Liverpool L3 5RF

E-mail: maya.wardeh@liverpool.ac.uk

Supplementary Note 1 – Host-pathogen interactions data and network construction

Non-human mammal-pathogen species interactions data

We extracted mammal-pathogen interactions from the Enhanced Infectious Diseases Database (EID2) (1). EID2 contains 4,799 species of mammals and 70,614 species in the taxa comprising most mammalian pathogens (bacteria= 18,249, fungi= 32,687, helminth= 6,305, protozoa= 3,768, viruses= 9,605).

EID2 utilises automated text and data-mining procedures to extract information on pathogens, their hosts and locations from two sources: 1) meta-data accompanying nucleotide sequences (hereafter sequences) published in GenBank (2,3) and 2) titles and abstracts of publications indexed in the PubMed database (4). To date, EID2 has extracted information from 71,076,379 sequences (and processed 100M+ sequences), and 8,643,203 PubMed titles and abstracts (TIABs).

For the purposes of this study we considered a mammal species to be host to a pathogen if at least four independent TIABs or one sequence reported an association between the host (and any of its subspecies) and the pathogen species (or any of its subspecies or strains).

Non-human domestication classification

We classified our mammalian hosts into three groups: wild mammals (N=1430), semi-domesticated mammals (N=102), and domesticated mammals (N=27).

We included the following mammals in our domesticated group: 13 ruminants (*Bison bonasus*, *Bos frontalis*, *Bos grunniens*, *Bos grunniens* x *Bos taurus*, *Bos indicus*, *Bos indicus* x *Bos taurus*, *Bos javanicus*, *Bos taurus*, *Bos taurus* x *Bos indicus*, *Bubalus bubalis*, *Bubalus carabanensis*, *Capra hircus*, and *Ovis aries*); 4 *Tylopoda* (*Camelus bactrianus*, *Camelus dromedaries*, *Lama glama* and *Lama pacos*); 3 carnivores (*Canis lupus familiaris*, F*elis catus* and *Vulpes vulpes*); 3 Perissodactyla (*Equus asinus*, *Equus asinus* x *caballus* and *Equus caballus*); and 1 of each of the following: Insectivora (*Atelerix albiventris*), Lagomorpha (*Oryctolagus cuniculus*), rodents (*Cavia porcellus*), and Suina (*Sus scrofa*).

We removed three domesticated rodent species from this study: ***Mus musculus, Rattus norvegicus*** and **R*attus rattus*.** These species are often associated with lab research in human (and other species) diseases, comprising approximately 95% of animal species used in research (5,6). Whilst EID2 has the capability of detecting and isolating laboratory generated host-pathogen associations (e.g. cell-lines, laboratory study publications); it cannot discriminate unlabelled associations (particularly in the genetic sequences meta-data). Therefore, we chose to remove these three species to avoid any contamination of our zoonoses definition.

Our semi-domesticated group included 35 ruminants, 31 carnivores, 22 rodents, 3 Diprotodontia, 3 Perissodactyla, 2 Tylopoda, 2 Proboscidea, and 1 of each: Dasyuromorphia, Pilosa, Sirenia and Suina.

Network statistics

To calculate the p-values for the network statistics listed in Table 1 (manuscript), we performed 1000 permutation on each of networks, whereby we shuffled the non-human mammalian species-level host-pathogen interactions extracted from EID2, and then re-generated the networks from the shuffled list and re-computed all statistics. The p-value listed in Table 1 (manuscript) is the number of times the statistics calculated for the permuted networks were at least as **extreme** as the ones observed in the original networks, divided by the number of permutations.

E-I Index

Given a categorical node attribute describing mutually exclusive groups, the E-I index represents a ratio of external to internal edges. We chose **order** as our categorical attribute and calculated E-I index at both group level (i.e. order), and global level (i.e. whole network). A positive group level E-I index indicates a tendency of the species in the order to share pathogens with species outside their order (i.e. extrovert). A negative EI index indicates a tendency to share pathogens within the group's order (i.e. introvert). Global level E-I index characterises the whole network in terms of bounded-ness and closure of its sub-groups (in terms of order). Order-level p-values was calculated from the 1000 permutations listed above, as the number of times the order-level E-I index in the shuffled networks was at least as **extreme** as the ones observed in the original networks, divided by the number of permutations.

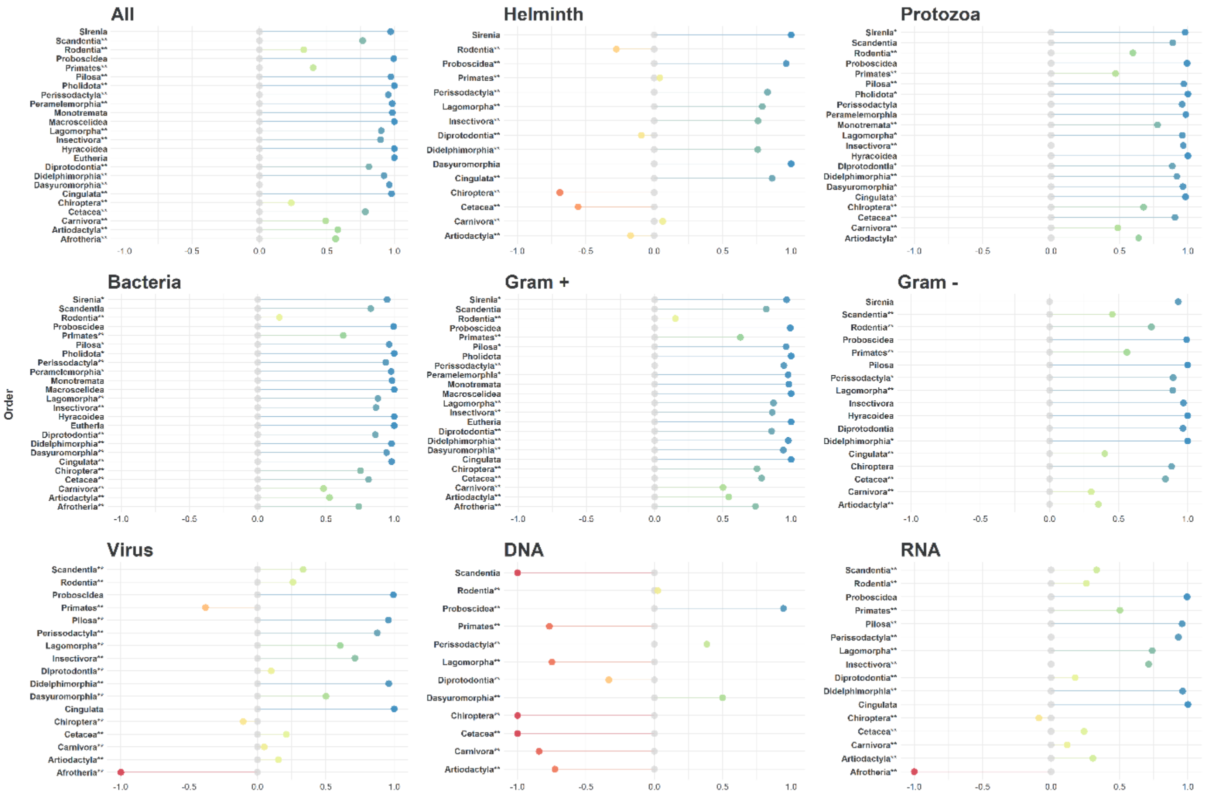

Figure SN1-1 – Order level EI-index. * next to the order name indicates permutation test p-value<=0.05, ** indicates p-value<=0.01.

References

1. Wardeh M, Risley C, Mcintyre MK, Setzkorn C, Baylis M. Database of host-pathogen and related species interactions, and their global distribution. Sci Data [Internet]. 2015;2.
2. Benson DA, Cavanaugh M, Clark K, Karsch-Mizrachi I, Lipman DJ, Ostell J, et al. GenBank. Nucleic Acids Res. 2013 Jan;41(Database issue):D36-42. http://www.ncbi.nlm.nih.gov/pubmed/23193287
3. Bethesda (MD): National Library of Medicine (US) NC for BI. GenBank [Internet] [Internet]. 1982. https://www.ncbi.nlm.nih.gov/nucleotide/
4. Bethesda (MD): National Library of Medicine (US). PubMed [Internet] [Internet]. 1946. https://www.ncbi.nlm.nih.gov/pubmed
5. Annual Statistics of Scientific Procedures on Living Animals, Great Britain 2017 [Internet]. https://assets.publishing.service.gov.uk/government/uploads/system/uploads/attachment_data/file/724611/annual-statistics-scientific-procedures-living-animals-2017.pdf
6. A Global View of Animal Experiments 2014 1 Executive Summary [Internet]. http://www.nationsonline.org/oneworld/countries_by_area.htm.

Supplementary Note 2 – Centrality measures

Centrality Measures

Degree & Eigenvalue based centrality measures: In general, degree centralities assess the importance of a node based on its reachability within a network. These measures provide a description of network connectivity based on the individual components.

Measure	Definition	Assumption/interpretation
**Degree centrality (DC)** (1)	$\mathrm{DC}(i) = K(i) = \sum_j a_{ij}$	Degree quantifies immediate risk of host species spreading or receiving pathogens. Host species with high degree may either: 1) pose high risk of pathogen transmission to many other host species, or 2) be at high risk of contracting pathogens from many other host species.
**Strength centrality (SC)** (2)	$SC(i) = K_w(i) = \sum_j w_{ij}$	Host species with high strength centrality values either: 1) share many pathogens with few hosts, or 2) share fewer pathogens with many hosts.
**Opsahl degree centrality (ODC)** (3)	$ODC(i) = K^{\alpha}(i) = K(i) \times \left[\frac{K^{w}(i)}{K(i)}\right]^{\alpha}$	Incorporates both the overall weights of the edges and the number of links to neighbouring nodes.
**Katz centrality (KC)** (4,5) / **Weighted Katz centrality (WKC)**	$KC^{k}(i) = x_i^k = \alpha \sum_j a_{ij}x_j + \beta$<br>$x = (I - \alpha A)^{-1}\beta$<br>Where A is the adjacency /weighted adjacency matrix of the network with eigenvalues λ. The parameter β controls the initial centrality and α < 1/λmax.	Katz centrality quantified the importance of a host within a network by measuring the number of immediate neighbours the host has, and also all other species reachable through these immediate neighbours. Connections made with distant neighbours are, however, penalized by an attenuation factor α. **In this study we computed α as the reciprocal of the first two eigenvalues** (5).
**Page rank centrality (PRC)** (6) / **Weighted Page rank centrality (WPRC)**	$C_{PR}(i) = (1-d) + d\left(\frac{C_{PR}(t_1)}{C(t_1)} + \cdots + \frac{C_{PR}(t_n)}{C(t_n)}\right)$<br>Where $d \in [1,0]$ is a damping factor, C(i) = number of edges from node i, and t_i = number of nodes pointing towards i.	PageRank is an adjustment of Katz centrality by which the more links a node attracts, the more important it is perceived.

Table SN2-1 – Degree & Eigenvalue centrality measures used in our analysis.

Distance based measures: Betweenness and Closeness define the flow pathways, which have been shown to be important in spreading pathogens across species(7,8). Nodes with high values of these metrics may act as bridges, connecting one part of a network to another that would otherwise be sparsely or not connected at all, favouring the spreading of disease agents across the entire network (3,9).

Measure	definition	Assumption/interpretation
**Closeness centrality (CC)** (10)	$CC(i) = \left[\sum_j^N d(i,j)\right]^{-1}$	Closeness is the inverse sum of shortest distances to all other host species from a focal host.
**Weighted Closeness centrality (WCC)** (3)	$d^w(i,j) = \left[\frac{1}{w_{ih}} + \cdots + \frac{1}{w_{hj}}\right]$<br>$WCC(i) = \left[\sum_j^N d^w(i,j)\right]^{-1}$	Weighted variation of closeness, by which edges encompassing larger number of pathogens are considered shorter than those containing fewer pathogens.

**Opsahl closeness centrality (OCC)** (3)	$d^{w\alpha}(i,j) = \left[\frac{1}{w_{ih}{}^{\alpha}} + \cdots + \frac{1}{w_{i}j^{\alpha}}\right]$ $OCC(i) = \sum_{j}^{V} \frac{1}{d^{w\alpha}(i,j)}$	Extends the shortest path algorithm by taking into consideration the number of intermediary hosts. ODC considers both the number of intermediary hosts (nodes) and the edges' weights (number of pathogens).
**Betweenness Centrality (BC)** (1)	$BC(i) = \sum_{j<k, j\neq k\neq i \in V} \frac{\sigma_{jk}(i)}{\sigma_{jk}}$ σ_{jk}= the number of shortest paths between nodes j and k. $\sigma_{jk}(i)$= the number of shortest paths that pass through node i out of σ_{jk}.	Betweenness centrality quantifies the number of times a host species acts as a bridge along the shortest path between two other hosts. It thus describes the importance of that node as an intermediary between different parts of the network. Hosts with betweenness > 0 act as bridges, connecting one part of a network to another that would otherwise be sparsely connected or not connected at all.
**Weighted Betweenness Centrality (WBC)** (11)	$WBC(i) = \sum_{j<k, j\neq k\neq i \in V} \frac{\sigma_{jk}^{w}(i)}{\sigma_{jk}^{w}}$	Weighted variation of betweenness. The more pathogens are shared between two nodes, the stronger of the flow between them.
**Opsahl Betweenness Centrality (OBC)** (3)	$OBC(i) = \sum_{j<k, j\neq k\neq i \in V} \frac{\sigma_{jk}^{w\alpha}(i)}{\sigma_{jk}^{w\alpha}}$	Extends betweenness by combining both the number of intermediary hosts (nodes) and the edges' weights (number of pathogens).

Table SN2-2 – Distance based centrality measures used in our analysis.

Principle component (PCA) and correlation analyses

We performed a Principle Component Analysis (PCA) on the full array of centrality measures, across the nine networks included in the study, to identify which measures contained the most relevant information and can effectively identify the most influential host species in each network. As illustrated in figure SN-1, the profile of the distance to the centre of the plot is different for each type of pathogen. Table SN2-3 shows the mean contribution of each centrality measure to the first five principle components of PCA analyses performed on each network. From this table it is evident that ODC has contributed the most to the first principle component, whereas OBC had the most contribution to the second principle component of our analyses. Figure SN2-2 show the clustering of our selected measures across the networks. The analysis was performed using the R package *FactoMineR* (12).

Additionally we performed correlation analyses on centrality measure in our networks. Table SN2-4 and figure SN2-3 show the results of these analyses.

	Dim.1	Dim.2	Dim.3	Dim.4	Dim.5
DC	8.40	2.77	2.94	7.03	4.23
SC	9.12	0.38	3.58	1.70	1.13
ODC	**9.13**	0.46	3.43	2.01	1.45
PRC	8.42	1.20	4.10	10.00	3.65
WPRC	8.61	1.73	3.85	1.38	5.13
CC	5.96	12.22	11.47	5.00	1.63
WCC	7.55	2.50	6.78	15.28	12.15
OCC	7.16	7.00	8.04	7.70	6.03
BC	5.58	16.81	6.75	1.94	4.05
WBC	5.72	10.66	7.95	8.72	24.03
OBC	3.19	**23.00**	17.67	9.40	24.16
KC	6.94	5.00	9.89	12.57	4.68
WKC	8.81	1.14	4.36	3.85	1.60
II	5.42	15.14	9.19	13.40	6.10

Table SN2-3 – Mean contribution of each centrality measure to the first 5 principle components of PCA analyses performed on the networks included in our study.

	SC	ODC	PRC	WPRC	CC	WCC	OCC	BC	WBC	OBC	KC	WKC	II
**DC**	0.92	0.98	0.95	0.86	0.79	0.78	0.8	0.57	0.62	0.36	0.9	0.88	**0.81**
**SC**		0.98	0.92	0.97	0.69	0.84	0.78	0.72	0.7	0.55	1	0.99	**0.66**
**ODC**			0.95	0.94	0.75	0.83	0.81	0.66	0.68	0.46	0.97	0.96	**0.74**
**PRC**				0.94	0.69	0.76	0.74	0.67	0.72	0.45	0.9	0.88	**0.74**
**WPRC**					0.6	0.79	0.71	0.78	0.76	0.6	0.96	0.95	**0.59**
**CC**						0.81	0.94	0.4	0.46	0.23	0.67	0.66	**0.88**
**WCC**							0.95	0.57	0.61	0.38	0.84	0.83	**0.70**
**OCC**								0.50	0.54	0.31	0.77	0.76	**0.82**
**BC**									0.96	0.84	0.72	0.71	**0.31**
**WBC**										0.7	0.69	0.68	**0.38**
**OBC**											0.56	0.57	**0.16**
**KC**													**0.63**
**WKC**													**0.61**

Table SN2-4 – mean correlation of centrality measure across all networks.

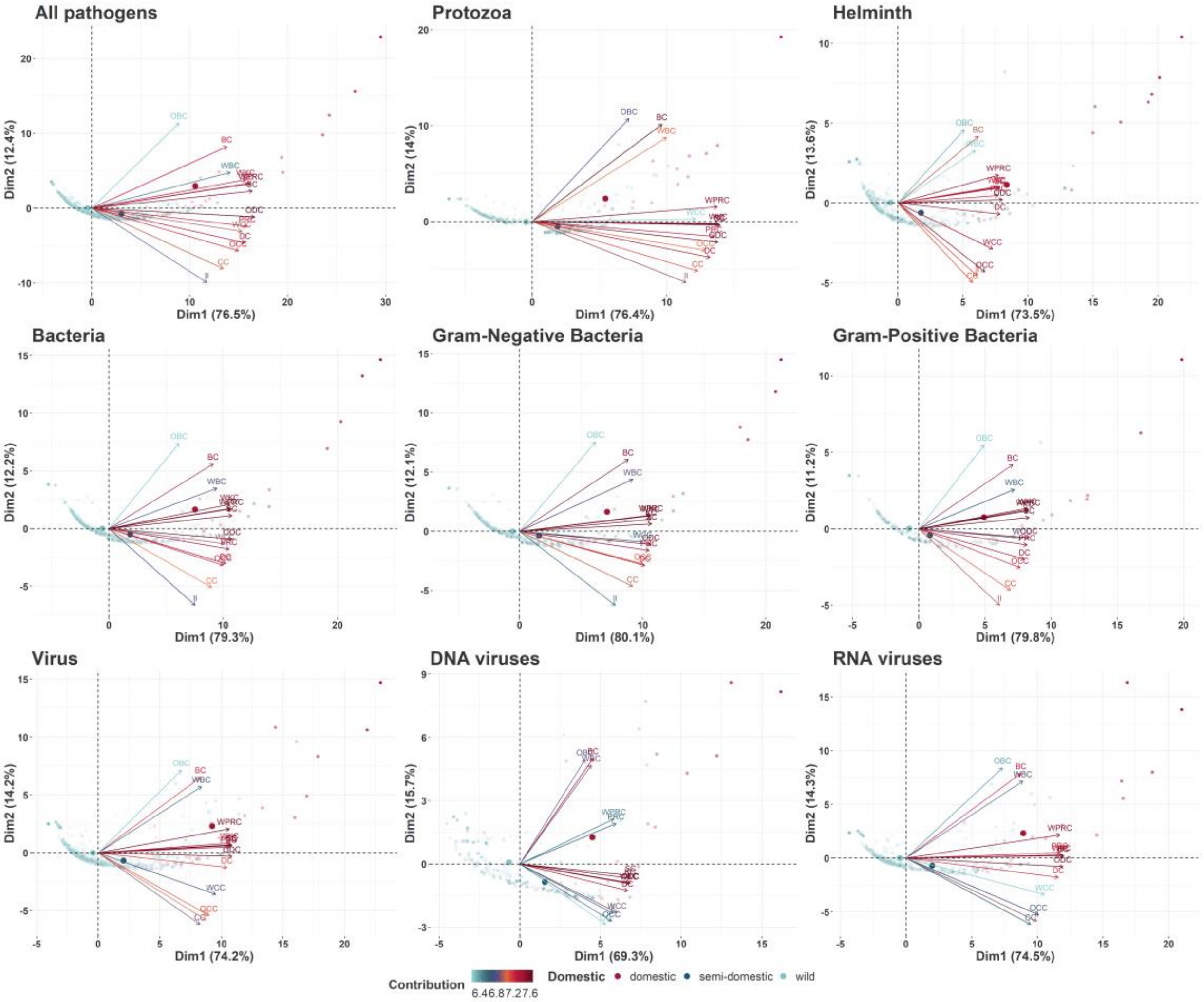

Figure SN2-1 - Biplot representation of the first two dimensions of PCA analyses performed on centrality measures from nine shared pathogen networks included in the study. In each plot, host species are shown as points, coloured by their domestication status, and centrality measures as vectors, coloured by their contribution.

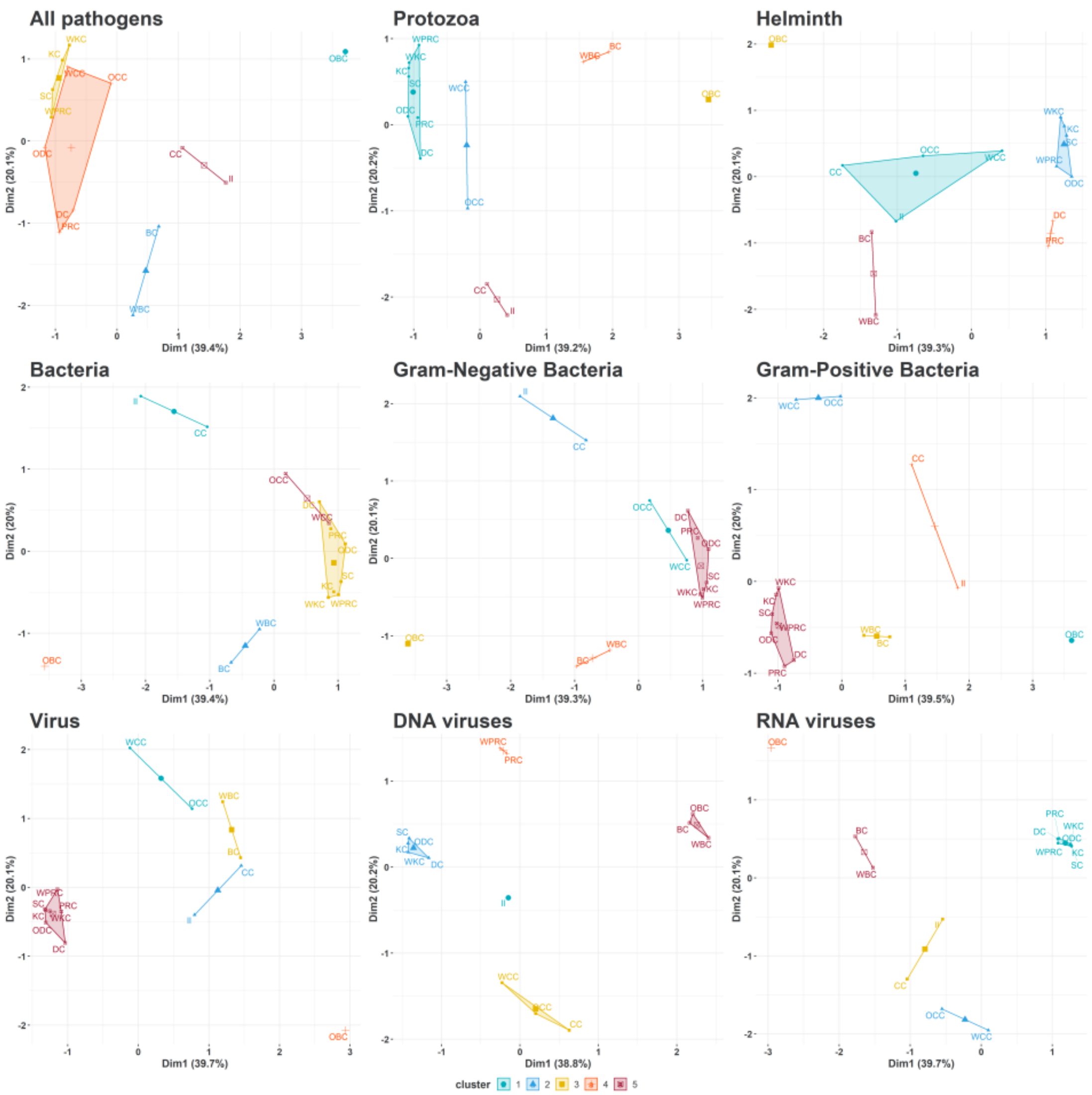

Figure SN2-2 – Clustering of centrality measures in each network based on their contribution in the principle component analysis. Clustering was performed using k-means (5 clusters, 1000 iterations). This clustering highlights how our measure Indirect Influence (II) clusters with closeness centralities.

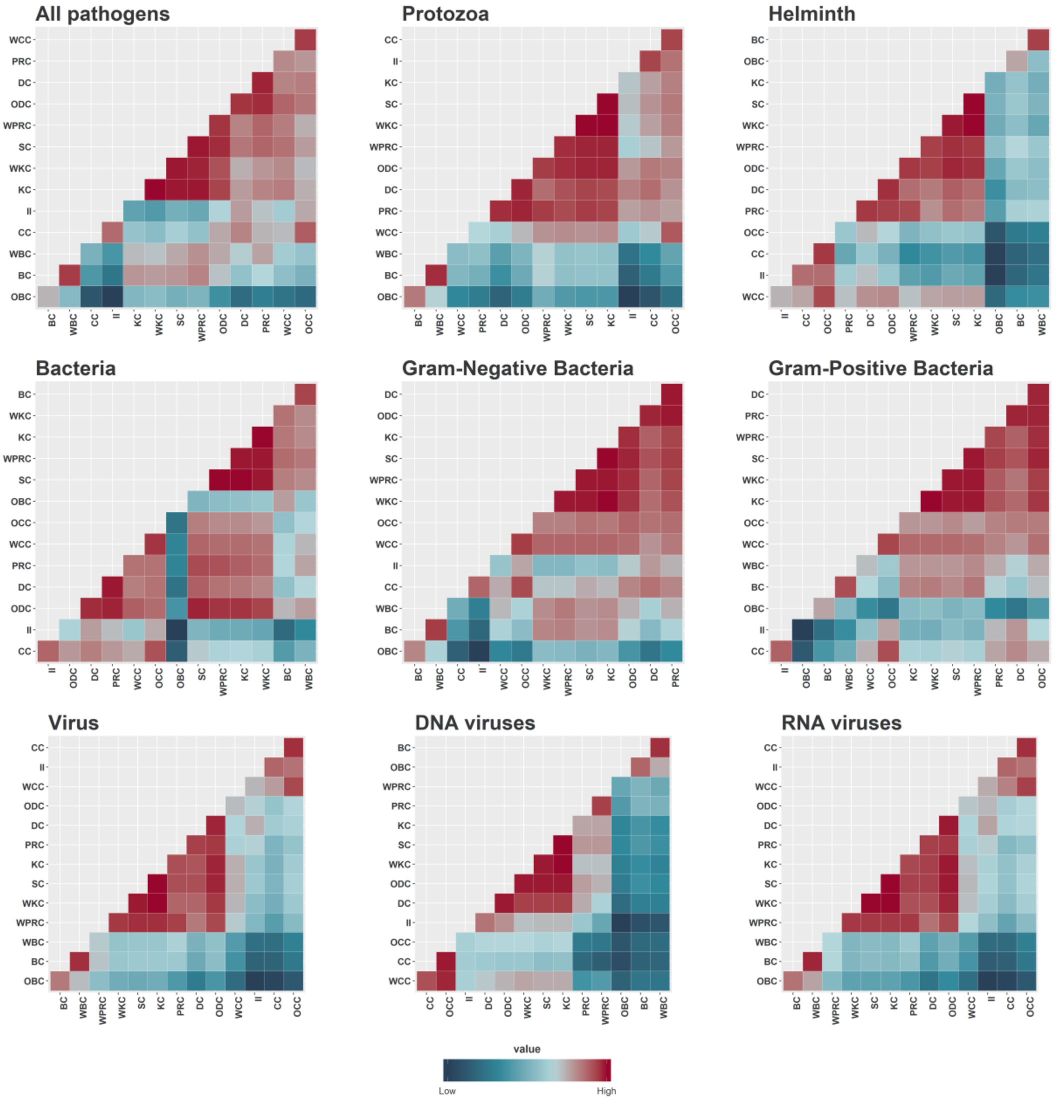

Figure SN2-3 – Correlation of centrality measures in each network. Centrality measures are re-ordered in network sub-plot using hierarchical clustering.

References

1. Freeman LC. A Set of Measures of Centrality Based on Betweenness. Sociometry. 1977;40(1):35. http://www.jstor.org/stable/3033543?origin=crossref
2. Barrat A, Barthélemy M, Pastor-Satorras R, Vespignani A. The architecture of complex weighted networks. Proc Natl Acad Sci U S A. 2004 Mar 16;101(11):3747–52. http://www.ncbi.nlm.nih.gov/pubmed/15007165
3. Opsahl T, Agneessens F, Skvoretz J. Node centrality in weighted networks: Generalizing degree and shortest paths. Soc Networks [Internet]. 2010;32(3):245–51.
4. Katz L. A new status index derived from sociometric analysis. Psychometrika [Internet]. 1953;18(1):39–43.
5. Sharkey KJ. Localization of eigenvector centrality in networks with a cut vertex. Phys Rev E. 2019 Jan 10;99(1):012315. https://link.aps.org/doi/10.1103/PhysRevE.99.012315
6. Brin S, Page L. The anatomy of a large scale hypertextual Web search engine. Comput Networks ISDN Syst [Internet]. 1998;30(1/7):107–17.
7. Kolaczyk E. Statistical analysis of network data [Internet]. Springer series in Statistics. 2009. 397 p. http://link.springer.com/content/pdf/10.1007/b98971.pdf
8. Borgatti SP. Centrality and network flow. Soc Networks [Internet]. 2005;27(1):55–71.
9. Newman MEJ. The Structure and Function of Complex Networks. SIAM Rev. 2003;45(2):167–256.

http://arxiv.org/abs/cond-mat/0303516
10. Freeman LC. Centrality in social networks conceptual clarification. Soc Networks [Internet]. 1978;1(3):215–39.
11. Newman MEJ. Spread of epidemic disease on networks. Phys Rev E - Stat Physics, Plasmas, Fluids, Relat Interdiscip Top [Internet]. 2002;66(1).
12. Husson F, Josse J, Le S, Maintainer JM. Package “FactoMineR” Title Multivariate Exploratory Data Analysis and Data Mining. 2018; http://factominer.free.fr

Supplementary Note 3 – Construction of ensemble models and their performance metrics

Ensembles to explain and predict centrality and influence measures

We developed a series of ensembles to investigate whether centrality in networks of shared-pathogens between mammalian hosts can be explained by these hosts' traits, phylogeny and relation to their neighbours in networks. We choose six learners to form the base models of our ensembles (Table SN3-1). We used R packages *Caret* (1,2) and *caretEnsemble* (3) to tune and train all our base models. We provided search grids for each bae learners to minimise Root Mean Square Error (RMSE) of the base learners and the ensembles based on 10-fold cross validation (100 repeats). Table SN3-2 lists mathematical frameworks of the metrics used to measure the performance of our models.

We utilised a greedy approach to construct our ensembles using the *caretEnsemble* (3) package. Each ensemble computed a weighted average of the predictions of its best-performing constituent learners. The performance of a learner is measured against a metric chosen at the training stage, and the weights are optimised using greedy algorithm to maximise or minimise our chosen metrics. Our ensembles to explain centrality were optimised to reduce RMSE. The greedy algorithm makes greedy choices at each step to ensure that the objective metric is optimised (minimising RMSE). The greedy algorithm has only one shot to compute the optimal solution (i.e., it never goes back and reverses any decision). The exact weights chosen varied with each fold of each training dataset. This dynamic nature allows the greedy ensemble to be tuned to perform well for each problem.

Model	Caret method/ R package	Tuning parameters
Stochastic Gradient Boosting	gbm /gbm	Number of trees, interaction depth (splits performed on each tree), shrinkage and minimum observation in node.
Support Vector Machines with Radial Basis Function Kernel	svmRadial/ kernlab	Sigma (smoothing variable) and cost.
k-Nearest Neighbors	knn/knn	Maximum number of neighbours.
Decision trees/CART	rpart/ rpart	complexity parameter
Random Forest	ranger/ ranger	number of variables to possibly split at in each node, minimal node size, and splitting rule (*gini* for classification & for regressions)
Lasso and Elastic-Net Regularized Generalized Linear Models (glmnet)	glmnet/ glmnet	The elastic-net mixing parameter (alpha) and lambda

Table SN3-1 – Base learners used in our ensemble models.

Measure	Formula	Meaning
preliminaries	y_i is the true (observed) value for instance i of the input data.<br>$\hat{y}_i$ is the predicted value.<br>n is total number of observations (data points).<br>k is the number of predictors in model.	
R²	$R^2 = 1 - \frac{\sum_{i=1}^{n}(\hat{y}_i - y_i)^2}{\sum_{i=1}^{n}(y_i - mean(y))^2}$	Coefficient of determination. R^2 provides a "goodness of fit" measure for the predictions to the observations. Values of R^2 ranges from 0 (no fit) to 1 (perfect fit).
Adjusted R²	$R^2_{adj} = 1 - [\frac{(n-1)(1-R^2)}{n-k-1}]$	Adjusted R^2 modifies R^2 for the number of predictors in the model. The adjusted R^2 increases only if the new term improves the model more than would be expected by chance. It decreases when a predictor improves the model by less than expected by chance. Values of adjusted R^2 ranges from 0 (no fit) to 1 (perfect fit).
RMSE	$RMSE = \frac{\sqrt{\sum_{i=1}^{n}(\hat{y}_i - y_i)^2}}{n}$	Root Mean Square Error (RMSE) is the standard deviation of the residuals (prediction errors).
NRMSE	$NRMSE = \frac{RMSE}{mean(y)}$	Normalised RMSE
MAE	$MAE = \frac{1}{n}\sum_{i=1}^{n}\lvert\hat{y}_i - y_i\rvert$	Mean Absolute Error (MAE), describes the typical magnitude of the residuals.
NMAE	$NMAE = \frac{MAE}{mean(y)}$	Normalised MAE

Table SN3-2 – Measures utilised to assess the performance of our 10-fold cross validated regression ensembles and their base components.

Ensembles to predict mammalian reservoirs of zoonoses, and to explain number of zoonoses shared with mammalian hosts

Using similar methodology to the one listed above. We developed two ensemble pipelines to answer two questions: 1) which mammals are more likely to harbour zoonotic pathogens? And 2) can the number of zoonoses be explained by centrality and/or host traits? Our ensembles comprised the same set of learners listed in table SN3-1.

Our ensembles to answer the first question (a classification problem) were optimised to maximise the Area under the ROC curve (AUC); and their constituent models were also trained to maximise their AUCs. We additionally computed a comprehensive set of metrics of these ensembles and their component models (table SN3-3) using 10-fold cross validation (100 repeats).

Our ensembles to answer the second question were constructed, tuned and assessed similarity to the ones discussed in the previous section.

<table>
<tr><td>Confusion matrix</td><td colspan="2"><table><tr><td></td><td colspan="2">Truth</td></tr><tr><td>Predicted</td><td>1</td><td>0</td></tr><tr><td>1</td><td>A</td><td>B</td></tr><tr><td>0</td><td>C</td><td>D</td></tr></table></td></tr>
<tr><td>Measure</td><td>Formula</td><td>Meaning</td></tr>
<tr><td>Sensitivity (recall)</td><td>A /(A + C)</td><td>Sensitivity is the percentage of actual 1’s that were correctly predicted. It indicates the percentage of 1s that was covered by the model.</td></tr>
<tr><td>Specificity</td><td>D/(B+D)</td><td>Specificity is the percentage of 0s that were correctly predicted</td></tr>
<tr><td>Precision</td><td>A/(A+B)</td><td>Percentage of accurate predictions of the model</td></tr>
<tr><td>AUC</td><td>Area Under the ROC Curve</td><td>Model's true performance considering all possible probability cut-offs</td></tr>
<tr><td>KS</td><td>Max(Cumulative% 1's - Cumulative% 0's)</td><td>KS statistic is the maximum difference between the cumulative percentage of responders or 1's (cumulative true positive rate) and cumulative percentage of non-responders or 0's (cumulative false positive rate).</td></tr>
<tr><td>F1-score</td><td>F1 Score = (2 * Precision * Recall) / (Precision + Recall)</td><td>A combination of Precision and Recall</td></tr>
<tr><td>TSS</td><td>sensitivity+ specificity-1</td><td>True skill statistics, provides a balance between correctly predicting the 1's and 0's</td></tr>
</table>

Table SN3-3 – Measures utilised to assess the performance of our 10-fold cross validated classification ensembles and their base components. Shaded rows are not included in the main text, but are used to calculate the model performance metric listed.

References

1. from Jed Wing MKC, Weston S, Williams A, Keefer C, Engelhardt A, Cooper T, et al. caret: Classification and Regression Training [Internet]. 2018. https://cran.r-project.org/package=caret
2. Kuhn M. Building Predictive Models in *R* Using the **caret** Package. J Stat Softw. 2008 Nov 10;28(5):1–26. http://www.jstatsoft.org/v28/i05/
3. Deane-Mayer ZA, Knowles JE. caretEnsemble: Ensembles of Caret Models [Internet]. 2016. https://cran.r-project.org/package=caretEnsemble

Supplementary Note 4 – Ensemble pipeline to explain centrality in networks of shared pathogens

Performance metrics

Network **Opsahl degree**

	R^2	Adjusted R^2	RMSE	NRMSE	MAE	NMAE
Overall	**0.868 [0.771, 0.971]**	**0.858 [0.732, 0.969]**	**39.6 [14.5, 112]**	**0.384 [0.130, 0.531]**	**25.3 [10.3, 71.2]**	**0.252 [0.085, 0.339]**
All	0.904 [0.893 - 0.917]	0.900 [0.890 - 0.914]	109.747 [104.49 - 115.19]	0.384 [0.366 - 0.403]	70.27 [67.25 - 73.03]	0.246 [0.235 - 0.256]
Bacteria	0.864 [0.842 - 0.882]	0.854 [0.83 - 0.872]	71.672 [66.88 - 76.25]	0.367 [0.342 - 0.390]	47.81 [45.32 - 50.35]	0.245 [0.232 - 0.258]
Gram -	0.835 [0.799 - 0.86]	0.801 [0.757 - 0.831]	33.442 [31.13 - 36.34]	0.377 [0.351 - 0.41]	24.77 [23.14 - 26.62]	0.28 [0.261 - 0.300]
Gram +	0.877 [0.857 - 0.891]	0.866 [0.844 - 0.881]	58.068 [54.75 - 61.36]	0.317 [0.299 - 0.335]	39.13 [37.24 - 41.14]	0.213 [0.203 - 0.224]
Helminth	0.839 [0.814 - 0.87]	0.826 [0.798 - 0.859]	29.64 [27.64 - 31.78]	0.516 [0.481 - 0.553]	19.09 [18.01 - 20.31]	0.332 [0.313 - 0.353]
Protozoa	0.968 [0.962 - 0.972]	0.966 [0.959 - 0.97]	37.667 [34.39 - 40.71]	0.133 [0.122 - 0.144]	24.528 [23.24 - 26.12]	0.087 [0.082 - 0.093]
Virus	0.864 [0.845 - 0.88]	0.855 [0.836 - 0.873]	43.695 [41.11 - 46.42]	0.422 [0.397 - 0.449]	28.67 [27.04 - 29.93]	0.277 [0.261 - 0.289]
DNA	0.782 [0.748 - 0.818]	0.746 [0.705 - 0.788]	14.93 [13.69 - 15.88]	0.415 [0.380 - 0.441]	10.647 [9.78 - 11.34]	0.296 [0.272 - 0.315]
RNA	0.88 [0.863 - 0.897]	0.871 [0.853 - 0.889]	38.98 [35.95 - 41.91]	0.393 [0.363 - 0.423]	24.42 [23.12 - 25.84]	0.246 [0.233 - 0.261]

Table SN4-1 - Performance metrics of our ensemble models to explain Opsahl Degree centrality in networks of shared pathogens between non-human mammals. Values in brackets indicate 95% confidence intervals of the metric. These intervals were obtained from the 100 runs (per pathogen taxa) of 10 fold cross validation performed.

Network	**Opsahl Betweenness**					
	**R^2**	**Adjusted R^2**	**RMSE**	**NRMSE**	**MAE**	**NMAE**
Overall	**0.687 [0.433, 0.923]**	**0.658 [0.364 , 0.916]**	**326 [75.4 , 1219]**	**0.785 [0.540, 1.240]**	**212.0 [72.7, 662.0]**	**0.509 [0.386 , 0.738]**
All pathogens	0.713 [0.496 - 0.906]	0.703 [0.478 - 0.903]	1128.057 [789.377 - 1375.273]	1.13 [0.791 - 1.378]	625.633 [463.29 - 753.692]	0.627 [0.464 - 0.755]
Bacteria	0.688 [0.369 - 0.957]	0.664 [0.321 - 0.954]	330.249 [214.597 - 425.754]	0.959 [0.623 - 1.237]	216.685 [176.666 - 256.799]	0.629 [0.513 - 0.746]
Gram -	0.635 [0.409 - 0.835]	0.559 [0.286 - 0.801]	81.878 [67.11 - 98.218]	0.639 [0.524 - 0.767]	83.491 [67.962 - 97.872]	0.652 [0.531 - 0.764]
Gram +	0.722 [0.486 - 0.946]	0.698 [0.442 - 0.942]	246.227 [170.523 - 333.618]	0.836 [0.579 - 1.133]	177.657 [143.342 - 212.356]	0.603 [0.487 - 0.721]
Helminth	0.584 [0.405 - 0.801]	0.549 [0.355 - 0.784]	444.389 [333.531 - 581.973]	0.82 [0.615 - 1.073]	272.529 [228.633 - 321.855]	0.503 [0.422 - 0.594]
Protozoa	0.706 [0.535 - 0.868]	0.684 [0.5 - 0.858]	182.782 [153.071 - 216.286]	0.69 [0.578 - 0.816]	115.357 [99.061 - 138.035]	0.435 [0.374 - 0.521]
Virus	0.729 [0.541 - 0.897]	0.712 [0.514 - 0.891]	502.739 [405.111 - 628.682]	0.818 [0.659 - 1.023]	274.209 [241.677 - 307.853]	0.446 [0.393 - 0.501]
DNA	0.777 [0.529 - 0.962]	0.740 [0.45 - 0.955]	121.005 [87.909 - 145.904]	0.647 [0.47 - 0.78]	81.114 [66.814 - 94.11]	0.433 [0.357 - 0.503]
RNA	0.601 [0.436 - 0.848]	0.573 [0.397 - 0.838]	396.562 [309.141 - 480.075]	0.825 [0.643 - 0.999]	233.172 [198.255 - 263.467]	0.485 [0.413 - 0.548]

Table SN4-2 - Performance metrics of our ensemble models to explain Opsahl Betweenness centrality in networks of shared pathogens between non-human mammals. Values in brackets indicate 95% confidence intervals of the metric. These intervals were obtained from the 100 runs (per pathogen taxa) of 10 fold cross validation performed.

Network	**Indirect Influence**					
	**R^2**	**Adjusted R^2**	**RMSE**	**NRMSE**	**MAE**	**NMAE**
Overall	**0.873 [0.723, 0.962]**	**0.864 [0.666 0.959]**	**0.062 [0.039, 0.093]**	**0.123 [0.056, 0.162]**	**0.043 [0.023, 0.0618]**	**0.086 [0.033, 0.117]**
All	0.901 [0.892 - 0.91]	0.897 [0.888 - 0.907]	0.057 [0.054 - 0.059]	0.113 [0.109 - 0.117]	0.038 [0.037 - 0.04]	0.076 [0.073 - 0.079]
Bacteria	0.864 [0.846 - 0.884]	0.853 [0.834 - 0.875]	0.063 [0.058 - 0.066]	0.107 [0.098 - 0.113]	0.041 [0.039 - 0.043]	0.07 [0.066 - 0.073]
Gram -	0.742 [0.691 - 0.784]	0.688 [0.627 - 0.739]	0.09 [0.081 - 0.098]	0.153 [0.139 - 0.166]	0.06 [0.056 - 0.064]	0.102 [0.095 - 0.11]
Gram +	0.874 [0.853 - 0.895]	0.863 [0.84 - 0.886]	0.064 [0.059 - 0.069]	0.103 [0.095 - 0.111]	0.041 [0.039 - 0.043]	0.066 [0.062 - 0.068]
Helminth	0.867 [0.849 - 0.883]	0.856 [0.836 - 0.873]	0.062 [0.06 - 0.065]	0.156 [0.15 - 0.164]	0.046 [0.043 - 0.048]	0.115 [0.11 - 0.12]

Protozoa	0.96 [0.95 - 0.97]	0.957 [0.946 - 0.968]	0.041 [0.037 - 0.045]	0.059 [0.054 - 0.066]	0.023 [0.021 - 0.025]	0.034 [0.031 - 0.036]
Virus	0.872 [0.86 - 0.886]	0.865 [0.851 - 0.88]	0.062 [0.059 - 0.064]	0.129 [0.123 - 0.135]	0.044 [0.043 - 0.046]	0.093 [0.09 - 0.097]
DNA	0.858 [0.832 - 0.882]	0.834 [0.804 - 0.863]	0.063 [0.058 - 0.067]	0.141 [0.13 - 0.15]	0.047 [0.044 - 0.051]	0.106 [0.099 - 0.114]
RNA	0.89 [0.879 - 0.903]	0.882 [0.87 - 0.897]	0.062 [0.058 - 0.065]	0.123 [0.115 - 0.128]	0.044 [0.042 - 0.046]	0.086 [0.083 - 0.091]

Table SN4-3 - Performance metrics of our ensemble models to explain Indirect Influence in networks of shared pathogens between non-human mammals. Values in brackets indicate 95% confidence intervals of the metric. These intervals were obtained from the 100 runs (per pathogen taxa) of 10 fold cross validation performed.

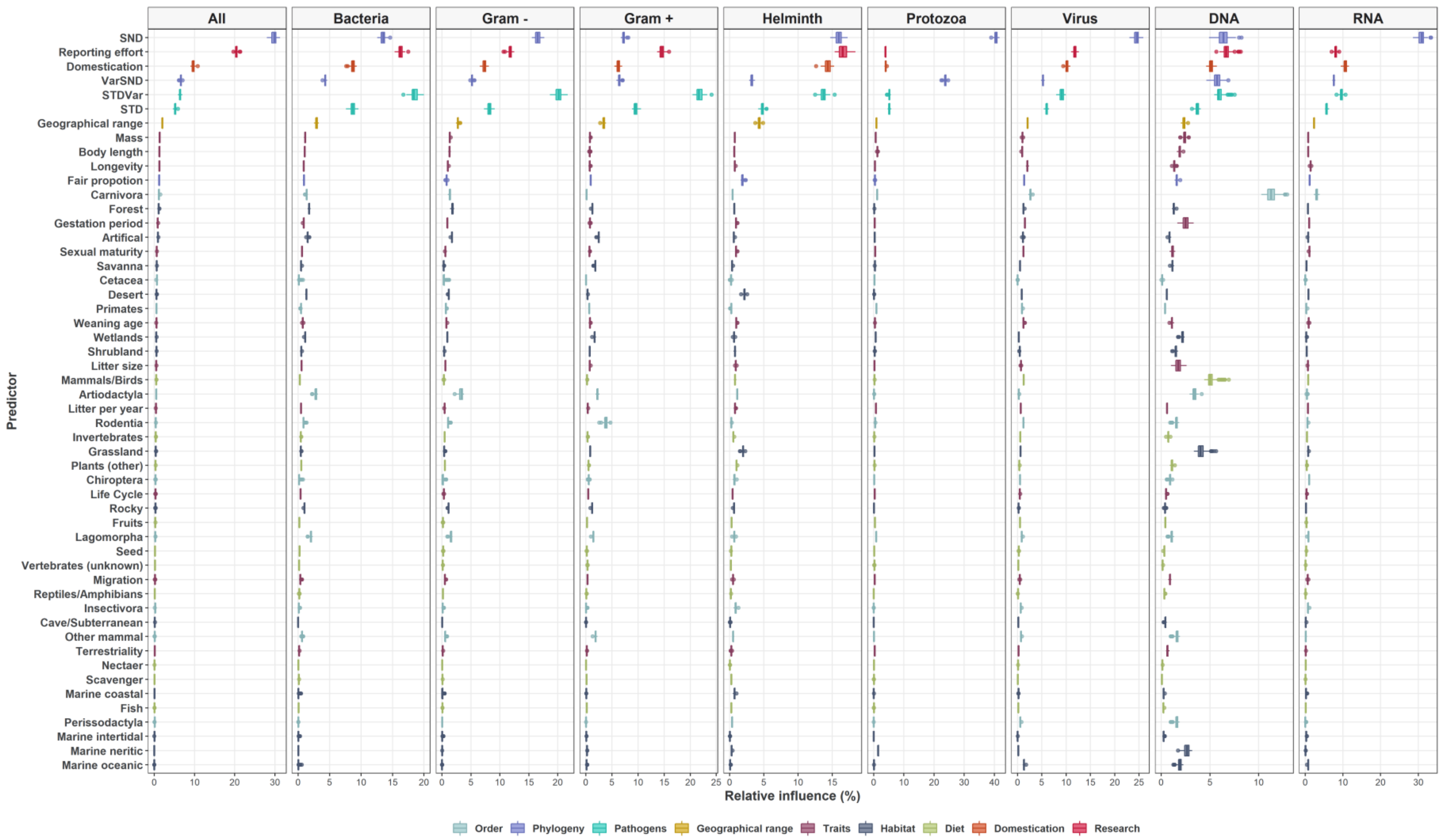

Figure SN4-2 – The relative influence all predictors included in our ensemble models to explain Opsahl degree centrality (ODC) in networks of shared pathogens amongst non-human mammals. Relative influence (variable importance) of predictors was calculated for each of the six base models and then averaged with weights (=contribution of models to the greedy ensemble) to produce final contribution. The predictors are coloured by their category.

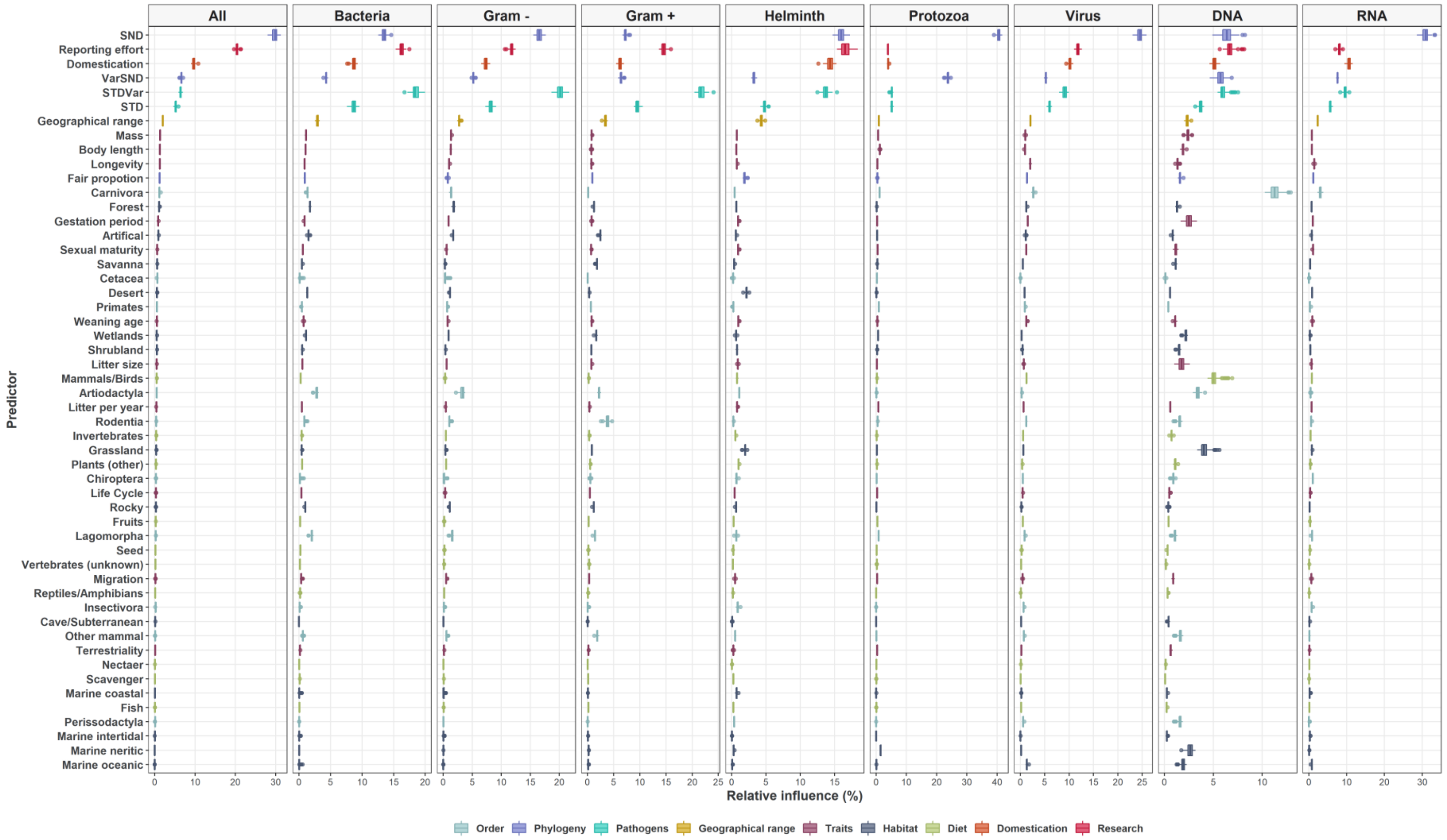

Figure SN4-3 – The relative influence all predictors included in our ensemble models to explain Opsahl betweenness centrality (OBC) in networks of shared pathogens amongst non-human mammals. Relative influence (variable importance) of predictors was calculated for each of the six base models and then averaged with weights (=contribution of models to the greedy ensemble) to produce final contribution. The predictors are coloured by their category.

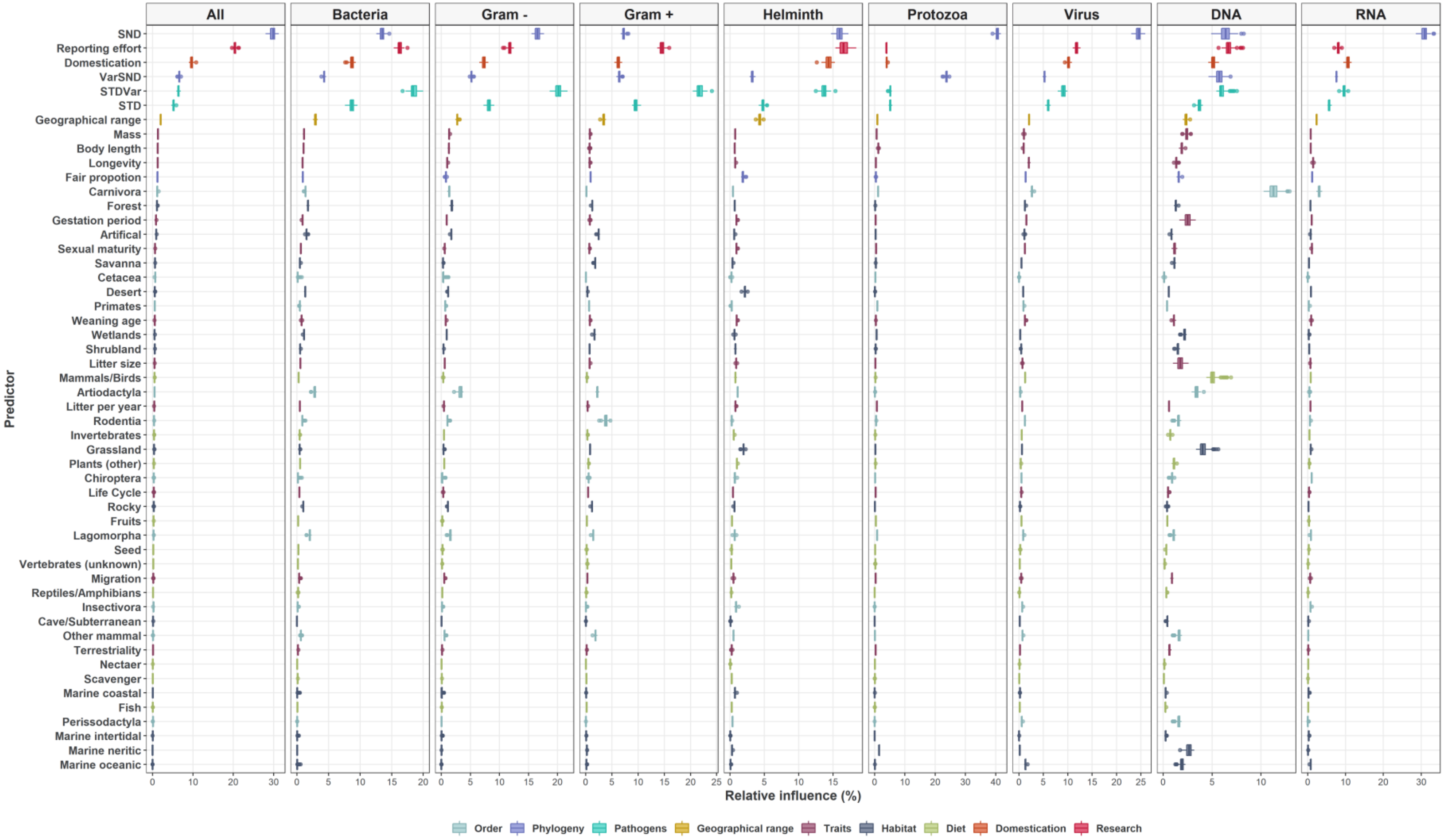

Figure SN4-4 – The relative influence all predictors included in our ensemble models to explain indirect influence (II) in networks of shared pathogens amongst non-human mammals. Relative influence (variable importance) of predictors was calculated for each of the six base models and then averaged with weights (=contribution of models to the greedy ensemble) to produce final contribution. The predictors are coloured by their category.

Supplementary Note 5 – Ensemble pipelines to predict mammalian reservoirs of zoonoses, and to explain number of zoonoses harboured by mammalian hosts

	AUC	TSS	KS	F1-score
Overall	0.966 [0.94, 1.000]	0.706 [0.455, 1.000]	74.138 [62.31, 100]	0.946 [0.778, 0.992]
Pathogen Taxa				
All	0.964 [0.958, 0.966]	0.672 [0.644, 0.722]	69.416 [66.82, 71.96]	0.946 [0.94, 0.950]
Bacteria	0.976 [0.959, 0.991]	0.535 [0.485, 0.739]	73.431 [62.06, 95.78]	0.978 [0.976, 0.985]
Gram -	0.990 [0.975, 1.000]	0.99 [0.25, 1.00]	99 [50, 100]	0.984 [0.983, 1.000]
Gram +	0.976 [0.963, 0.985]	0.496 [0.37, 0.639]	68.407 [57.59, 86.51]	0.976 [0.968, 0.980]
Helminth	0.965 [0.959, 0.974]	0.815 [0.788, 0.853]	81.73 [79.01, 85.65]	0.920 [0.909, 0.937]
Protozoa	0.996 [0.991, 0.999]	0.874 [0.822, 0.929]	90 [85.94, 97.78]	0.985 [0.981, 0.992]
Virus	0.946 [0.937, 0.958]	0.698 [0.657, 0.743]	70.94 [66.64, 75.15]	0.903 [0.889, 0.915]
DNA	0.952 [0.931, 0.970]	0.712 [0.625, 0.771]	76.72 [69.82, 84.08]	0.800 [0.732, 0.851]
RNA	0.953 [0.943, 0.964]	0.692 [0.635, 0.747]	72.32 [69.201, 76.36]	0.915 [0.905, 0.926]

Table SN5-1 - Performance metrics of our ensemble classification models to predict sharing of pathogens between mammals and humans. Reported values are median results of model runs (n=100), Values between brackets indicates 95% CI. KS values range from 0 to 100. Other values range from 0 to 1.

	R^2	Adjusted R^2	RMSE	NRMSE	MAE	NMAE
Overall	0.892 [0.778, 0.943]	0.887 [0.757, 0.942]	1.353 [0.687, 4.549]	0.675 [0.389, 1.17]	0.744 [0.39, 2.319]	0.368 [0.228, 0.66]
Pathogen Taxa						
All	0.934 [0.899, 0.954]	0.932 [0.897, 0.954]	4.209 [3.697, 4.848]	0.71 [0.624, 0.818]	1.913 [1.807, 2.381]	0.323 [0.305, 0.402]
Bacteria	0.891 [0.842, 0.94]	0.886 [0.835, 0.937]	3.404 [2.653, 4.423]	0.709 [0.553, 0.921]	1.712 [1.5, 1.986]	0.357 [0.313, 0.414]
Gram -	0.916 [0.886, 0.948]	0.912 [0.881, 0.945]	1.63 [1.341, 2.165]	0.478 [0.393, 0.635]	0.929 [0.829, 1.072]	0.272 [0.243, 0.314]
Gram +	0.828 [0.718, 0.902]	0.809 [0.687, 0.891]	2.901 [2.268, 3.92]	0.763 [0.597, 1.031]	1.746 [1.534, 2.236]	0.459 [0.404, 0.588]
Helminth	0.908 [0.877, 0.932]	0.903 [0.871, 0.929]	1.323 [1.135, 1.48]	0.706 [0.605, 0.789]	0.688 [0.623, 0.751]	0.367 [0.332, 0.401]
Protozoa	0.9 [0.864, 0.923]	0.896 [0.858, 0.92]	0.833 [0.726, 0.936]	0.406 [0.354, 0.456]	0.483 [0.442, 0.516]	0.236 [0.215, 0.251]
Virus	0.884 [0.845, 0.913]	0.88 [0.84, 0.91]	1.299 [1.152, 1.433]	0.685 [0.607, 0.756]	0.742 [0.696, 0.799]	0.391 [0.367, 0.421]
DNA	0.827 [0.764, 0.875]	0.812 [0.743, 0.864]	0.724 [0.629, 0.799]	1.124 [0.977, 1.241]	0.408 [0.368, 0.442]	0.633 [0.571, 0.686]
RNA	0.873 [0.827, 0.897]	0.868 [0.821, 0.893]	0.996 [0.932, 1.075]	0.574 [0.537, 0.62]	0.637 [0.594, 0.67]	0.367 [0.343, 0.387]

Table SN5-2 - Performance metrics of our ensemble regression models to explain number of pathogens shared between mammals and humans. Reported values are median results of model runs (n=100), Values between brackets indicates 95% CI.

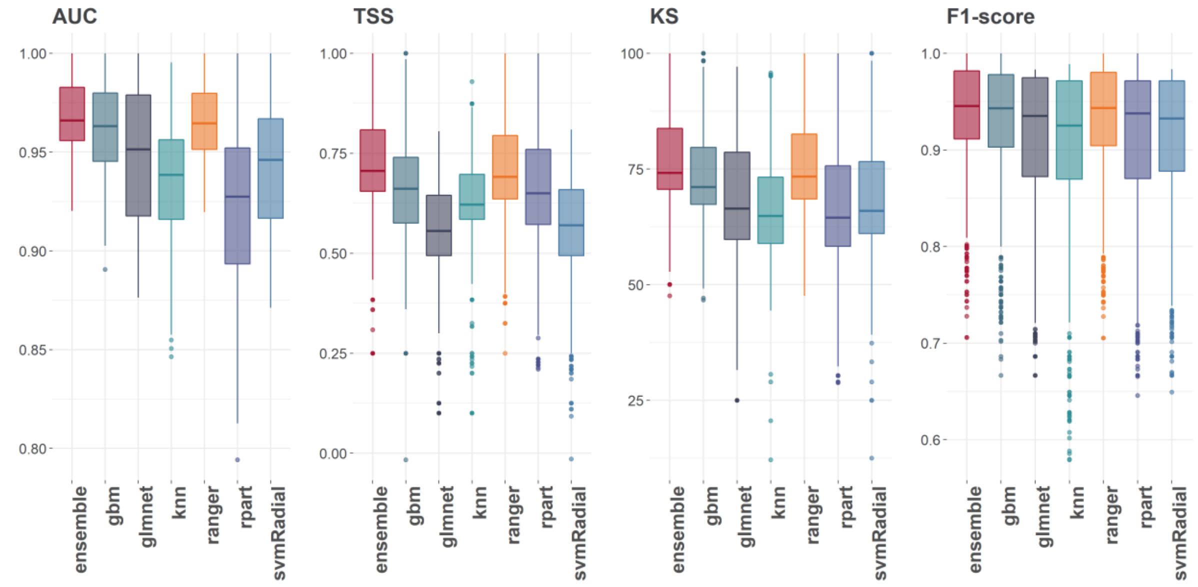

Figure SN5-1 – Performance metrics of our ensemble models to predict mammalian reservoirs of zoonoses (red) and their base learners. Each panel illustrates one performance metric calculated for all runs of the models: 100 runs of 10-fold cross validation per component (including ensembles) per type of pathogen (6300 runs in total). Higher values indicate better performance across all metrics.

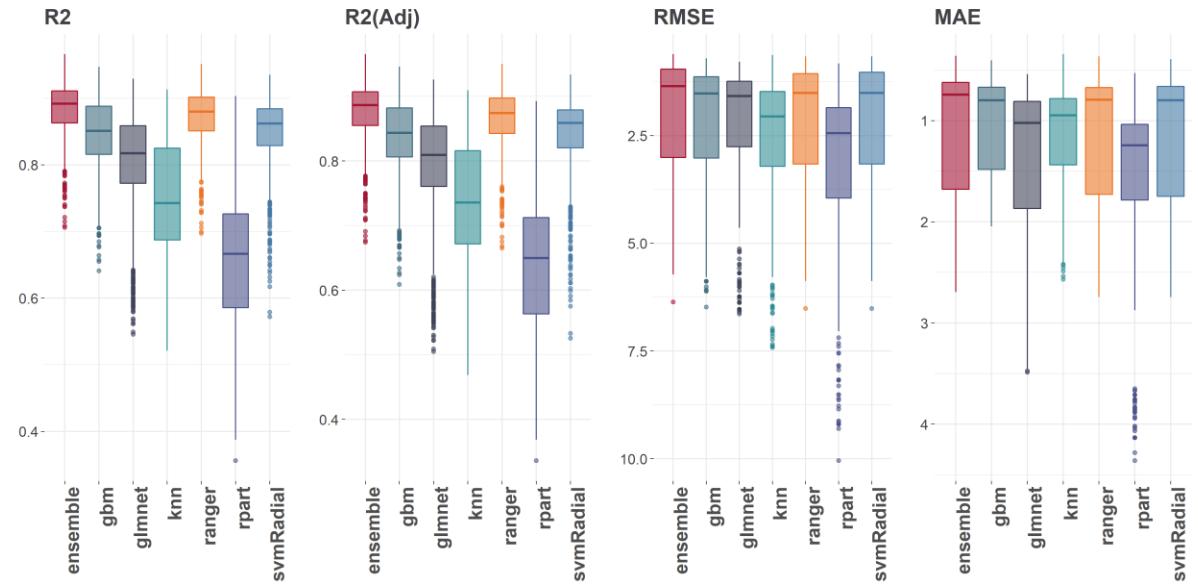

Figure SN5-2 – Performance metrics of our ensemble models to predict number of pathogens shared between mammals and humans (red) and their base learners. Each panel illustrates one performance metric calculated for all runs of the models: 100 runs of 10-fold cross validation per component (including ensembles) per type of pathogen (6300 runs in total). For R2 and adjusted R2 metrics higher values indicate better performance. For RMSE and MAE metrics lower values indicates better performance.

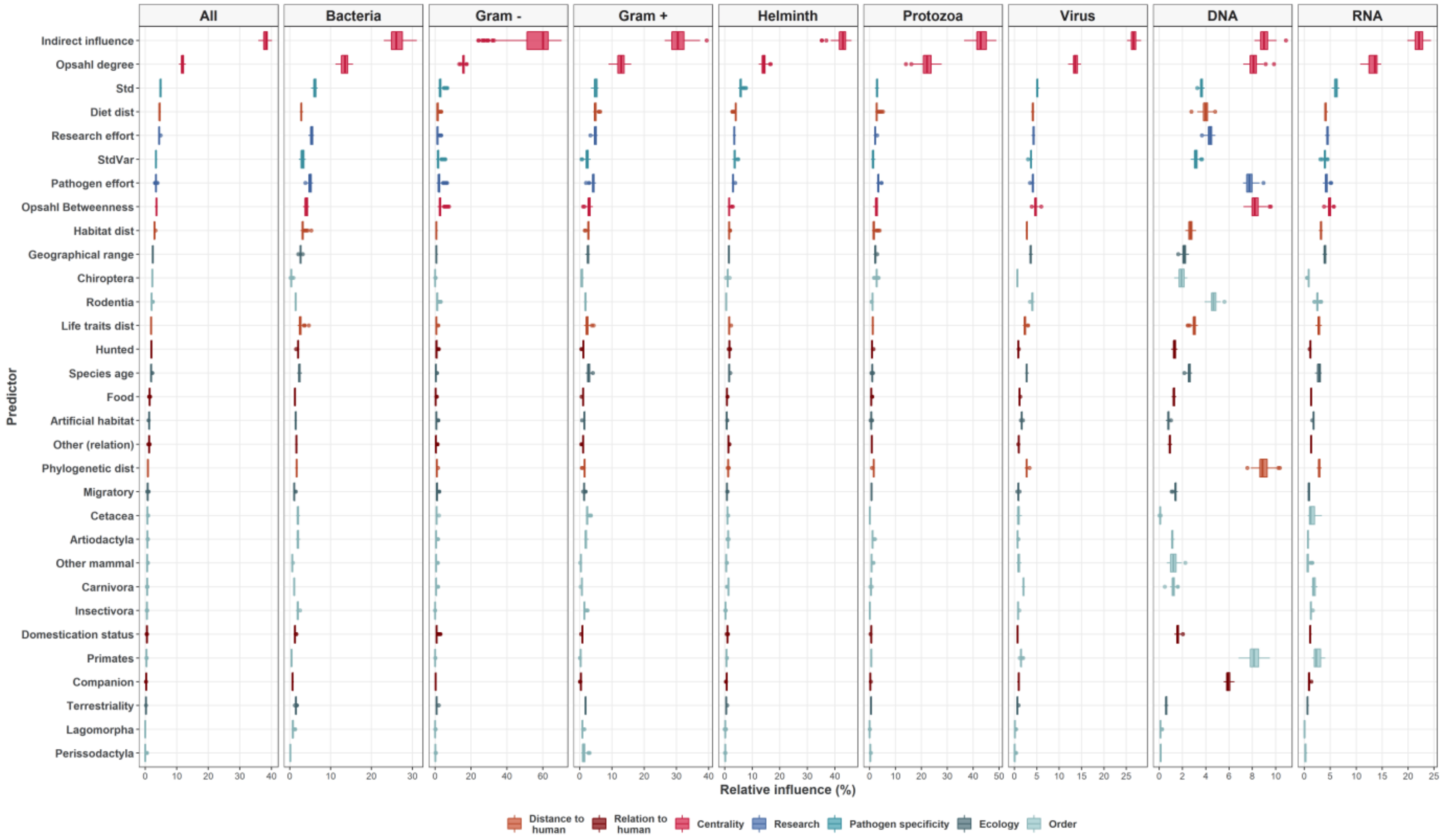

Figure SN5-3 – The relative influence of predictors included in our ensemble models to predict mammalian reservoirs of zoonoses. Relative influence (variable importance) of predictors was calculated for each of the six base models and then averaged with weights (=contribution of models to the greedy ensemble) to produce final contribution. Predictors are coloured by their category.

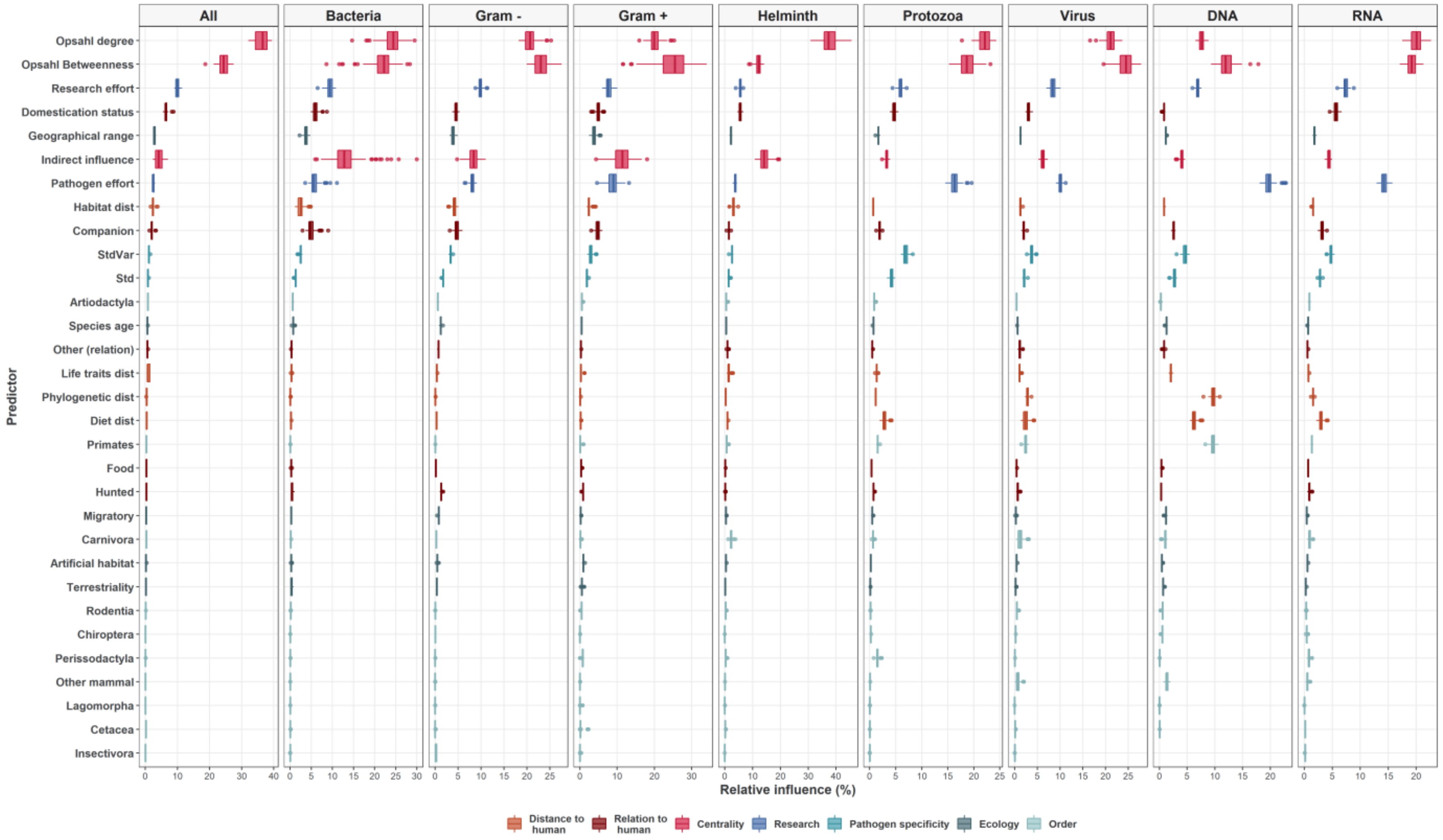

Figure SN5-4 – The relative influence of host order predictors included in our ensemble models to predict sharing of pathogens between mammals and humans. Relative influence (variable importance) of predictors was calculated for each of the six base models and then averaged with weights (=contribution of models to the greedy ensemble) to produce final contribution. Predictors are coloured by their category.
